# Microbial community structure and resistome dynamics on elevator buttons in response to surface disinfection practices

**DOI:** 10.3389/fpubh.2025.1593114

**Published:** 2025-05-30

**Authors:** Shanshan Ye, Shifu Peng, Xiaolei Wang, Jingjing Fan, Chenxue Zhu, Liye Huang, Ying Huang, Keping Cheng, Tingting Ni, Yuqing Qian, Xiaosong Wu, Yan Xu

**Affiliations:** ^1^Jiangsu Provincial Center for Disease Control and Prevention, Nanjing, Jiangsu, China; ^2^School of Public Health, Nanjing Medical University, Nanjing, Jiangsu, China; ^3^National Health Committee Key Laboratory of Enteric Pathogenic Microbiology, Nanjing, Jiangsu, China; ^4^Xuzhou Center for Disease Control and Prevention, Xuzhou, Jiangsu, China; ^5^Infection Control Department, The Second Hospital of Nanjing, Affiliated to Nanjing University of Chinese Medicine, Nanjing, China; ^6^Zhongda Hospital Affiliated to Southeast University, Nanjing, China; ^7^Jiangsu Provincial Medical Key Laboratory of Pathogenic Microbiology in Emerging Major Infectious Diseases, Nanjing, Jiangsu, China

**Keywords:** disinfectant, elevator buttons, antibiotic resistance, disinfectant resistance, metagenomics, mobile genetic elements

## Abstract

**Background:**

Disinfectants have been extensively used in public environments since the COVID-19 outbreak to help control the spread of the virus. This study aims to investigate whether disinfectant use influences the structure of bacterial communities and contributes to bacterial resistance to disinfectants and antibiotics.

**Methods:**

Using molecular biology techniques—including metagenomic sequencing and quantitative PCR (qPCR)—we analyzed the bacterial communities on elevator button surfaces from two tertiary hospitals, one infectious disease hospital, two quarantine hotels (designated for COVID-19 control), and five general hotels in Nanjing, Jiangsu Province, during the COVID-19 pandemic. We focused on detecting disinfectant resistance genes (DRGs), antibiotic resistance genes (ARGs), and mobile genetic elements (MGEs).

**Results:**

Significant differences were observed in the bacterial community structures on elevator button surfaces across the four types of environments. Quarantine hotels, which implemented the most frequent disinfection protocols, exhibited distinct bacterial profiles at the phylum, genus, and species levels. Both *α*-diversity (within-sample diversity) and *β*-diversity (between-sample diversity) were lower and more distinct in quarantine hotels compared to the other environments. The abundance of DRGs, ARGs, and MGEs was also significantly higher on elevator button surfaces in quarantine hotels. Notably, antibiotic-resistant bacteria (ARBs), including *Escherichia coli*, *Acinetobacter baumannii*, and *Pseudomonas aeruginosa*, were detected in all four settings.

**Conclusion:**

The structure of bacterial communities on elevator button surfaces varies across different environments, likely influenced by the frequency of disinfectant use. Increased resistance gene abundance in quarantine hotels suggests that disinfection practices may contribute to the selection and spread of resistant bacteria. Enhanced monitoring of disinfection effectiveness and refinement of protocols in high-risk environments such as hospitals and hotels are essential to limit the spread of resistant pathogens.

## 1 Introduction

In March 2020, the World Health Organization (WHO) officially declared the 2019 COVID-19 outbreak a global pandemic (1, 2). At this time, the WHO emphasized the importance of proper and consistent disinfection and environmental cleaning practices (3). Although effective vaccines against SARS-CoV-2—the virus responsible for COVID-19—have been developed (4), and some therapeutic advancements have been made (5), disinfection remains a critical strategy for eliminating viral and bacterial pathogens and reducing infection transmission in both household and community settings, as most microorganisms can be effectively killed by disinfectants (6–8). When used appropriately, disinfectants have been shown to lower microbial contamination and prevent infections (9). However, excessive use of disinfectants can result in significant environmental residue buildup (10), which not only threatens human health but also contributes to a range of environmental issues.

Disinfectants have been reported to impact biological communities (11). The large-scale use of disinfectants during the COVID-19 pandemic may have disrupted ecological balances between microorganisms and their hosts (12), thereby altering the structure of bacterial communities (13). As antimicrobial agents, disinfectants exert selective pressure that can promote the emergence of resistant cells (7) and studies have shown that repeated exposure may increase bacterial tolerance (13). Moreover, the overuse and misuse of disinfectants could contribute to the development of antibiotic resistance in bacteria (14).

Previous research on the negative effects of disinfectants has largely focused on municipal waste and natural environments (15, 16). However, during the COVID-19 pandemic, disinfection efforts were especially intensified in community and public spaces such as schools, office buildings, shops, hospitals, and quarantine hotels (17–19). Medical facilities and hotels—particularly those used for quarantine—often employed high-frequency, high-dose disinfection protocols to maximize efficacy and maintain health and safety. Elevators, as high-traffic public areas, are particularly vulnerable to microbial contamination (20). Elevator buttons are high-contact surfaces and potential carriers of pathogenic microorganisms (18). As a result, they have become key targets for disinfection. The structure of microbial communities plays a vital role in maintaining ecosystem balance and can serve as an indicator of environmental pollution and ecological health (21, 22). However, the widespread use of disinfectants during the COVID-19 pandemic may have influenced the microbial composition on elevator button surfaces. Because disinfectants have specific bactericidal spectra, they selectively inhibit or eliminate certain bacteria, while others are less affected. This selective action alters bacterial abundance and can disrupt the overall microbial structure (7).

Historically, research and practical efforts have primarily focused on the quality of disinfectants, proper preparation and storage methods, and potential bacterial contamination of disinfectant containers, along with the effectiveness of disinfection. However, growing attention is now being directed toward environmental risk factors, particularly the misuse and overuse of disinfectants. Frequent and high-dose applications may contribute to the development of increased bacterial tolerance to disinfectants (13). Bacteria can develop resistance to disinfectants by carrying disinfectant resistance genes (DRGs). If the concentration of commonly used disinfectants is not effective in killing bacteria, it may inadvertently promote the spread of bacteria.

In parallel, the widespread use of antibiotics has led to the emergence of antibiotic-resistant bacteria (ARBs), including strains resistant to three or more commonly used antibiotics—commonly referred to as multidrug-resistant (MDR) bacteria. Increasing evidence suggests a link between disinfectant use and the development of antibiotic resistance. Studies have shown that extensive use of quaternary ammonium compounds, a common class of disinfectants, may enhance bacterial resistance to antibiotics (23). Therefore, we hypothesize that the frequency and type of hospital-grade disinfectant applied to elevator buttons will significantly shape the resident bacterial community composition, and antibiotic resistome.

In this study, swab samples were collected from elevator buttons in general hospitals (tertiary hospitals), quarantine hotels, standard hotels, and an infectious disease hospital. Metagenomic analysis was employed to investigate the structure of bacterial communities and their resistance to both disinfectants and antibiotics.

## 2 Materials and methods

### 2.1 Site selection and sampling

**Figure 1:**
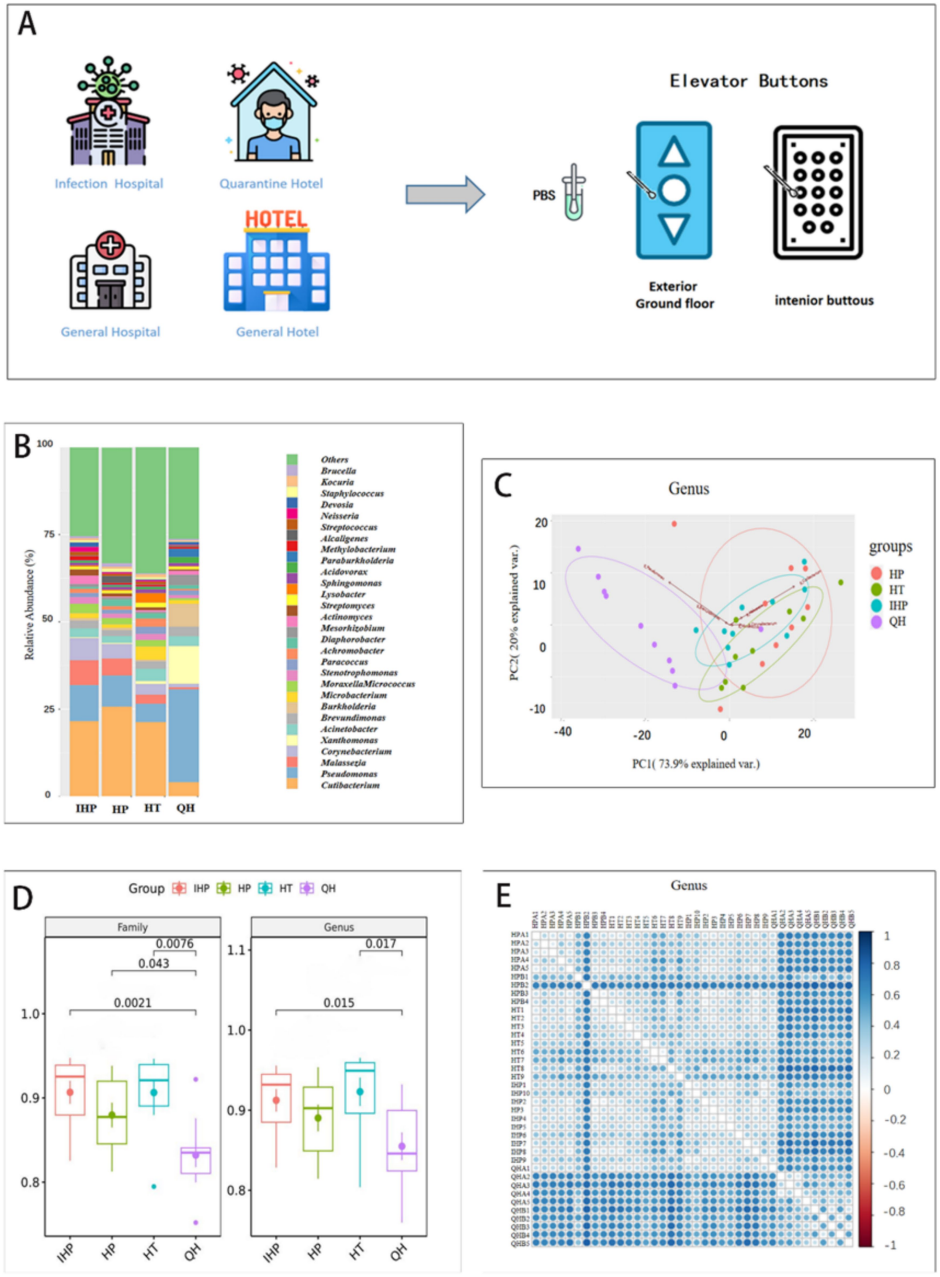
IHP, HP, HT, QH represent infectious disease hospital, general hospitals, general hotels, and quarantine hotels, respectively. **(A)** The elevator buttons surface was sampled in infectious disease hospital, quarantine hotels, general hospitals, and general hotels. **(B)** Distribution and relative abundance of the top 30 bacterial genera in four environments. **(C)** Principal component analysis of 30 genus-level bacterial communities based on metagenomes. The first two principal components (PC1 and PC2) explain 93.9% of the variance in the data. The different colors represent four different places. **(D)** A diversity of bacteria at genus and family levels in four environments. **(E)** *β* diversity indicates that lower values (indicated by lighter colors) occur in venues of the same type. HPA1-5 and HPB1-4 represent elevator samples from general hospitals. HT1-9 and IPP1-10 represent the elevator samples from general hotels and infectious disease hospital, respectively. QHA1-5 and QHB1-5 are from quarantine hotel.

As shown in Figure 1A, swab samples were collected between September and October 2022 from elevator buttons in two general hospitals, one infectious disease hospital, two quarantine hotels, and five general hotels in Nanjing, Jiangsu Province, China. In total, 41 elevator button samples were collected, of which 38 met the inclusion criteria and were selected for further analysis. Among these, 10 samples were from quarantine hotels, 9 from general hospitals, 10 from the infectious disease hospital, and 9 from general hotels.

Disinfection frequency varied by site. In general hospitals, the infectious disease hospital, and general hotels, elevator buttons were disinfected 4–8 times daily. In quarantine hotels, buttons were disinfected after each use, amounting to at least 20 times per day. Disinfectant types also differed: chlorine-based disinfectants at a concentration of 1,000 mg/L were used in infectious disease and general hospitals; 75% ethanol was used in general hotels; and both 75% ethanol and 1,000 mg/L chlorine-based disinfectants were applied in quarantine hotels. Prior to COVID-19, elevator buttons in most public settings were not routinely disinfected, based on site interviews and institutional policies.

The daily human traffic varied significantly across the four types of facilities included in the study. According to public or administrative records, the general hospitals experienced the highest volume, with approximately 5,000 visitors per day. In contrast, infectious disease hospitals saw about 500 visitors daily. General hotels accommodated around 200–300 guests per day, while quarantine hotels had the lowest occupancy, hosting approximately 100–150 individuals per day, with movement restrictions in place due to COVID-19 control measures.

The sampling protocol followed a previously established method (24). Briefly, for each elevator, one swab was used to sample the exterior “up” button on the ground floor (the most frequently used) and all interior buttons. Swabs were pre-moistened with sterile phosphate-buffered saline (PBS) for the initial collection. Samples were stored at −20°C or −80°C until DNA extraction and metagenomic analysis. All elevators remained in normal operation during the sampling period. A second round of sampling was conducted 2 days later, using pre-moistened swabs with a neutralizing sampling solution.

### 2.2 Sample processing and metagenomic sequencing

Total genomic DNA was extracted using the TIANGEN DP336 kit, following the manufacturer’s instructions. DNA concentration and purity were assessed using agarose gel electrophoresis and ultraviolet absorbance (ND1000, NanoDrop, Thermo Fisher Scientific Inc.), and the extracted DNA was stored at −20°C for subsequent analysis. Sequencing libraries were prepared using the TruSeq^®^ DNA PCR-Free Sample Preparation Kit (Illumina, United States), and unique index codes were added to each sample. Library quality was evaluated using a Qubit^®^ 2.0 Fluorometer (Thermo Scientific) and an Agilent Bioanalyzer 2,100 system. High-throughput sequencing was performed on the Illumina platform (150 bp paired-end reads), generating an average of 10 GB of clean data per sample.

### 2.3 Metagenome data processing

Paired-end reads were demultiplexed based on unique barcode and primer sequences. High-quality clean reads were obtained using a previously published protocol with stringent filtering criteria. Briefly, raw metagenomic reads were quality-filtered using Fastp v0.23.2 with certain parameters. Reads containing adapters or >5% ambiguous bases were removed. Assembly of the high-quality reads was carried out using MEGAHIT (version 1.0.6), and open reading frames (ORFs) were predicted using MetaGeneMark (version 3.38). Redundant sequences were removed through clustering, and representative genomes with over 90% completeness and less than 5% contamination were selected for downstream analysis.

Taxonomic classification of the ORFs was performed using the DIAMOND alignment tool against a microbial reference database. Antibiotic resistance genes (ARGs) were identified by aligning sequences to the Comprehensive Antibiotic Resistance Database (CARD) and were categorized into more than 20 antibiotic classes, including beta-lactams, aminoglycosides, phenicols, fluoroquinolones, sulfonamides, and combination therapies. A custom MGE database was constructed by integrating reference sequences from ISFinder, INTEGRALL, and ACLAME, comprising insertion sequences, integrons, transposons, and plasmid elements. Sequences were clustered at 95% identity using CD-HIT to remove redundancy. Only experimentally validated MGEs were retained, and known contaminant or host genomic elements were excluded based on NCBI annotations.

### 2.4 Quantification of DRGs, ARGs, and MGEs

Quantification methods followed previously published protocols (25). Briefly, the count data for disinfectant resistance genes (DRGs), antibiotic resistance genes (ARGs), and mobile genetic elements (MGEs) were normalized based on the 16S rRNA gene copy number. Abundance was expressed as the number of gene copies per 16S rRNA gene copy.

### 2.5 Microbiome data statistical analyses

Alpha diversity (*α*-diversity) was assessed using the Shannon index, calculated with relevant packages in R software (version 3.4.2). Differences in α-diversity between case and control groups were tested using the Wilcoxon Rank Sum Test. Beta diversity (*β*-diversity) was evaluated using Bray–Curtis distance, computed with the vegan package in R. Principal component analysis (PCA) was conducted to explore the internal structure and variation within the microbial community data. The Metastats method was used to identify statistically significant differences in taxonomic abundance across groups. A *p*-value of less than 0.05 was considered statistically significant in all analyses.

## 3 Results

### 3.1 Bacterial community structures

The bacterial community structure on elevator button surfaces in quarantine hotels differed notably from those in the other three settings. As shown in Figure 1B, at the genus level, *Pseudomonas*, *Xanthomonas*, and *Burkholderia* were the dominant genera in quarantine hotels. In contrast, *Cutibacterium* was predominant in the infectious disease hospital, general hospitals, and general hotels. At the family level, *Pseudomonadaceae* dominated the microbial community in quarantine hotels, whereas *Propionibacteriaceae* was dominant in the other three environments (Supplementary Figure S1). At the species level, *Xanthomonas campestris* was the dominant species in quarantine hotels, while *Cutibacterium acnes* was predominant at the other three sites (Supplementary Figure S2).

Principal Component Analysis (PCA) was used to assess the differences in bacterial community composition across sample types. Samples with more similar community structures are positioned closer together in the PCA plot. As shown in Figure 1C, at the genus level, the first two principal components accounted for 93.9% of the total variance and clearly separated the samples into four distinct groups. Notably, samples from quarantine hotels clustered separately from those of the other three environments, indicating a distinct microbial composition.

Alpha diversity (*α*-diversity), which reflects species richness within a single environment, was assessed using the Shannon index. As shown in Figure 1D, at the genus level, the Shannon index for quarantine hotels was lower than that of the infectious disease hospital, general hospitals, and general hotels, indicating reduced microbial diversity. Statistically significant differences in genus-level diversity were found between quarantine hotels and both the infectious disease hospital and general hotels. At the family level, the Shannon index for the infectious disease hospital was significantly lower than that of the other three environments. Significant differences were also observed between quarantine hotels and the other sites (infectious disease hospital, general hospitals, and general hotels).

Beta diversity (*β*-diversity), which evaluates differences in microbial composition between environments, further confirmed these distinctions. As shown in Figure 1E, the microbial communities in general hotels, general hospitals, and the infectious disease hospital were more similar to each other than to those in quarantine hotels. Analysis of Similarities (ANOSIM) based on Bray–Curtis dissimilarity revealed significant differences among groups (R = 0.394, *p* = 0.001), with greater variability observed within the quarantine hotel samples (Supplementary Figure S3). These findings suggest that microbial diversity in quarantine hotels was more variable compared to the other three environments.

### 3.2 Characterization of disinfectant resistance

Disinfectant resistance refers to the failure of disinfectants, at their standard concentrations and recommended contact times, to effectively kill or inhibit microorganisms. In this study, 44 known DRGs were examined, and 22 of these genes were detected across the collected samples. Notably, the abundance of DRGs was significantly higher in samples from quarantine hotels compared to those from the other three environments (data not shown).

**Figure 2:**
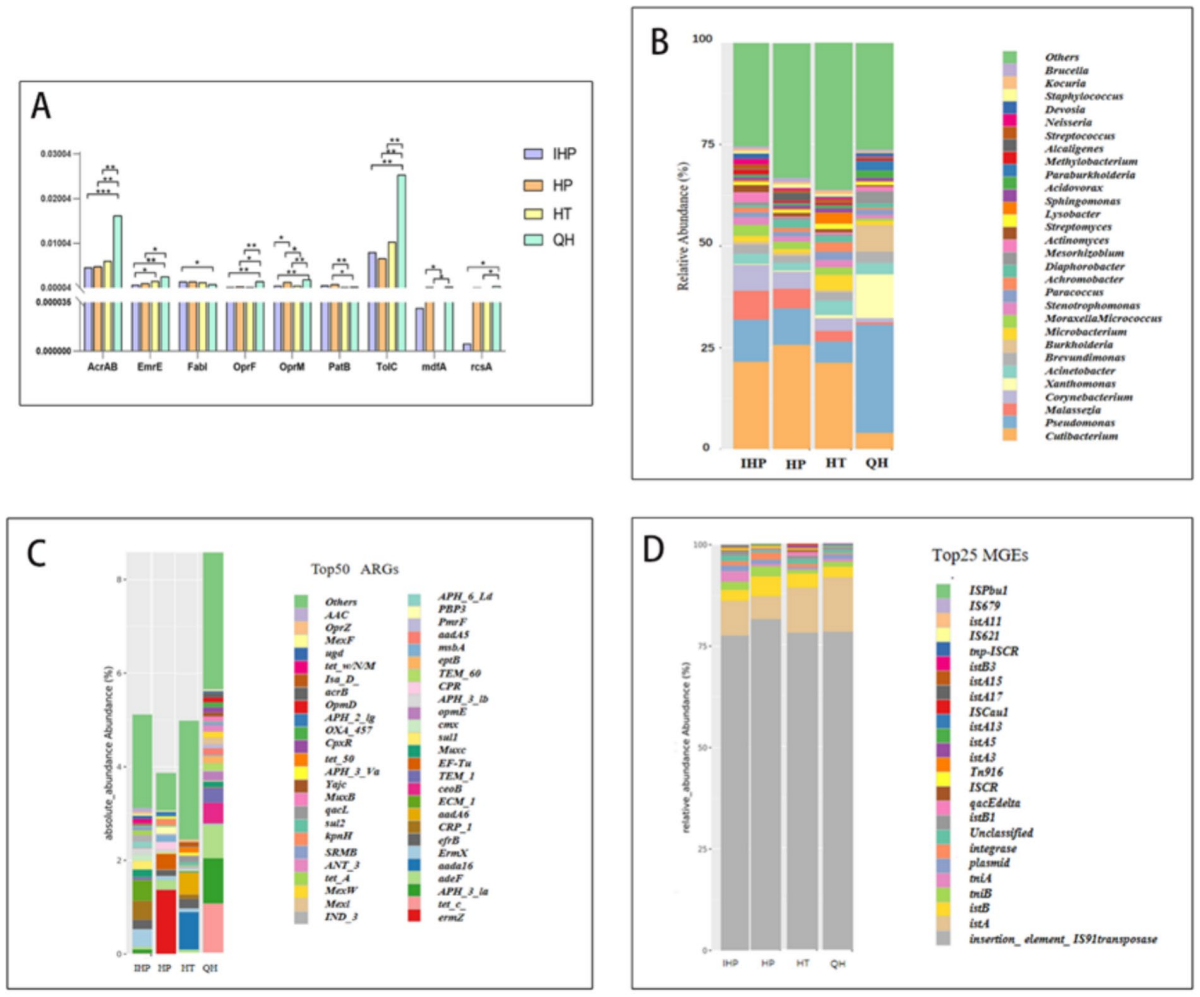
IHP, HP, HT, QH represent infectious disease hospital, general hospitals, general hotels, and quarantine hotels, respectively. **(A)** After Wilcoxon statistical test, the distribution of statistically significant disinfectant resistance genes in the two groups showed that * represented *p* < 0.05, ** represented *p* < 0.01, *** represented *p* < 0.001. **(B)** Absolute abundance of antibiotic resistance of different bacterial types in four environments. **(C)** Absolute abundance of the top 50 resistance genes in four environments. **(D)** Relative abundance of MGE in four environments.

As shown in Figure 2A, three transporter-encoding genes—*AcrAB*, *OprF*, and *TolC*—exhibited statistically significant differences in abundance between quarantine hotels and the other sites. These genes encode outer membrane channel proteins that contribute to resistance by facilitating the efflux of toxic substances, including disinfectants, from bacterial cells. In addition to these, other resistance genes identified included *qacA*, *qacB*, *qacE*, *qacJ*, *EmrE*, and *mdfA*, which are commonly associated with resistance to quaternary ammonium compounds (QACs) and other disinfectant agents (Figure 2B).

**Figure 3:**
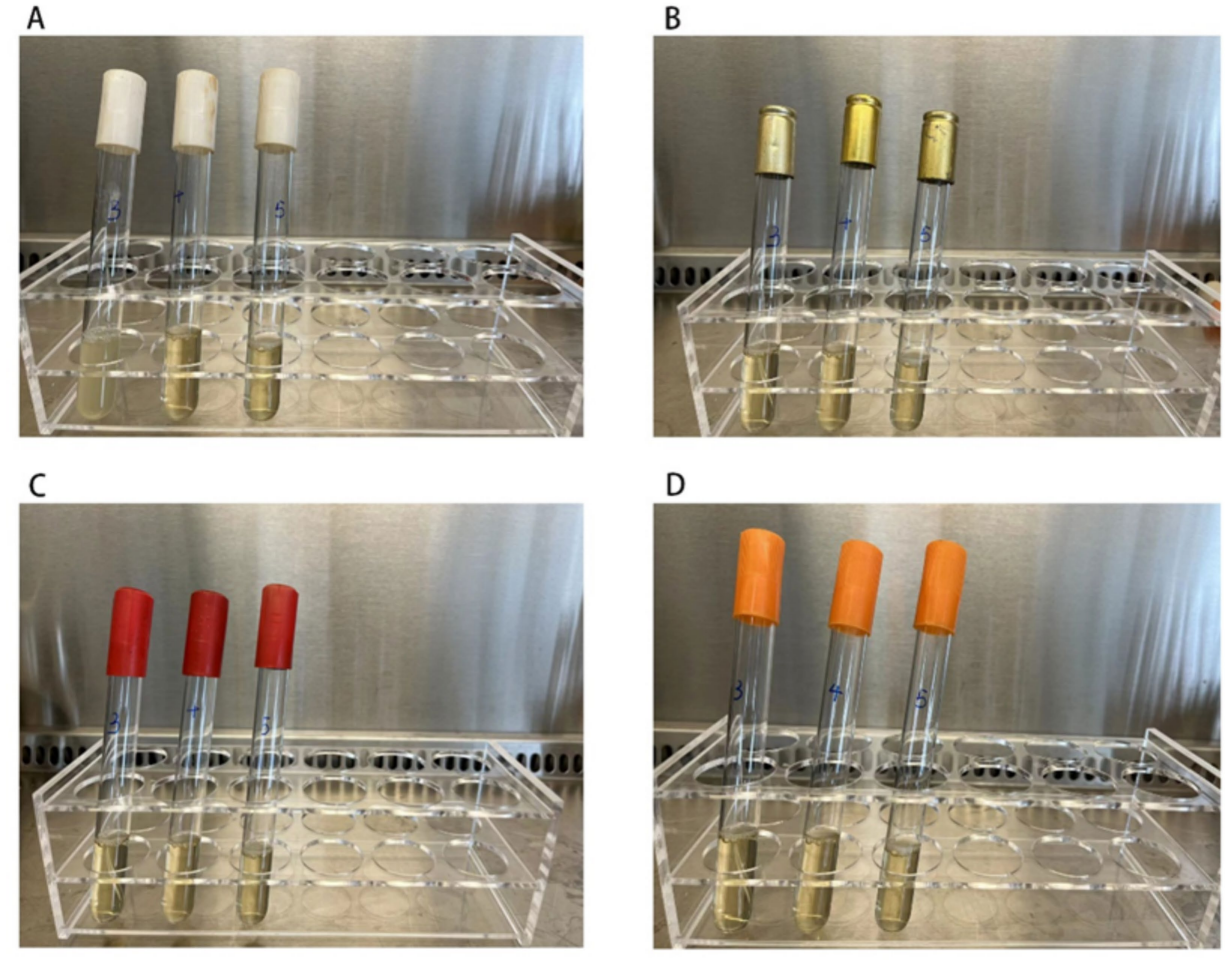
The figure shows the MBC experiment results of 4 strains of *Staphylococcus aureus*. The MBC value of *Staphylococcus aureus* isolated from the quarantine hotels was 250 mg/L, and the MBC value of *Staphylococcus aureus* isolated from the general hotels, infectious disease hospital, and general hospitals was 200 mg/L. The concentration of chlorine-containing disinfectant in 3 test tubes from left to right in each picture was increasing at 200 mg/L, 250 mg/L, and 300 mg/L, respectively. Turbidity indicates bacterial growth, while clear indicates no bacterial growth. **(A)** *Staphylococcus aureus* isolated from the surface of the quarantine hotels, turbidity only observed in the first tube. **(B–D)** Represents the MBC results of *Staphylococcus aureus* which isolated from the surface of the general hotels, of the general hospitals, and of the infectious disease hospital, no growth was observed these isolated *Staphylococcus aureus*.

Minimum inhibitory concentration (MIC) testing revealed that *Staphylococcus aureus* strains exhibited a MIC of 200 mg/L against chlorine-based disinfectants (Supplementary Figure S4). As shown in Figure 3, the minimum bactericidal concentration (MBC) of *S. aureus* isolated from quarantine hotels was 250 mg/L—higher than the MBCs observed in isolates from the other three environments. This finding suggests an elevated level of disinfectant tolerance in bacterial populations from quarantine hotels.

### 3.3 Characterization of antibiotic resistance

Pathogenic bacteria such as *Escherichia coli*, *Staphylococcus aureus*, and *Acinetobacter baumannii* were detected across all four environments, as shown in Figure 2B and Supplementary Figure S5. The abundance of antibiotic-resistant bacteria (ARB) was also significantly higher in quarantine hotels compared to the other three sites.

As shown in Figure 2C, samples collected from quarantine hotels exhibited the highest overall abundance of antibiotic resistance genes (ARGs) among the four environments studied. Specifically, the most abundant ARGs detected were *ermZ* in infectious disease hospitals, *ErmX* in general hospitals, *aadA16* in general hotels, and *tetC* in quarantine hotels. These findings suggest that quarantine hotels not only harbor more ARGs but may also serve as reservoirs for clinically relevant ARBs.

### 3.4 Characterization of MGEs in four places

Mobile genetic elements (MGEs) are DNA sequences capable of moving within and between genomes, and include transposons, plasmids, integrons, and phages. MGEs facilitate the horizontal transfer of genetic material, including resistance genes, thereby accelerating microbial evolution and the spread of antimicrobial resistance.

Among the four environments, quarantine hotels exhibited the highest overall abundance of MGEs (Supplementary Figure S6). As shown in Figure 2D, transposase genes and the insertion sequence *IS91* were the most prevalent MGEs detected. These elements likely play a significant role in the dissemination of both disinfectant and antibiotic resistance genes in the microbial communities found on elevator buttons in quarantine hotels.

## 4 Discussion

In this study, conducted during the COVID-19 pandemic, swab samples were collected from elevator button surfaces in two general hospitals, one infectious disease hospital, two quarantine hotels, and five general hotels. We analyzed the bacterial community structures present in these settings. *Streptococci* and *Pseudomonas* were commonly detected on the elevator button surfaces in both medical institutions and hotels, consistent with findings by Kandel et al. and Pereira et al. regarding surface-associated bacteria (24, 26).

Alpha-diversity analysis revealed that microbial species diversity on elevator buttons was higher in general hospitals, the infectious disease hospital, and general hotels compared to quarantine hotels. Similarly, beta-diversity (*β*-diversity) analysis showed that microbial communities in quarantine hotels were distinctly different from those in the other three environments. The bacterial communities on elevator buttons in quarantine hotels exhibited greater compositional variability, indicating a more heterogeneous microbial landscape. At the family, genus, and species levels, dominant bacterial taxa in quarantine hotels differed significantly from those in the other environments. For instance, *Pseudomonas syringae* was more abundant on elevator buttons in quarantine hotels, whereas *Propionibacterium acnes* was more prevalent on buttons in general hospitals, the infectious disease hospital, and general hotels.

These differences may be largely attributed to the varying use of elevators by different populations across settings. Prior research by Guo et al. suggests that patients with respiratory infections are more susceptible to airborne pathogens, which may influence microbial spread (27, 28). Another key factor is the type and frequency of disinfectant use. Quarantine hotels demonstrated the most frequent elevator disinfection practices. However, excessive disinfection may selectively eliminate more sensitive bacterial species, thereby altering the overall bacterial composition of surfaces (8). Here, we acknowledge that this was an observational field study, and multiple confounding variables—such as human microbiota differences, environmental ventilation, surface material, and user behavior—could influence microbial composition. While disinfectant practices were a major variable, our findings should be interpreted in the context of these co-existing factors.

A variety of DRGs were detected across the four environments examined in this study—general hospitals, an infectious disease hospital, quarantine hotels, and general hotels. Among these, the highest abundance of DRGs was found on the surfaces of elevator buttons in quarantine hotels. In particular, the genes *AcrAB*, *OprF*, and *TolC* were significantly more abundant in bacterial communities from quarantine hotel samples compared to those from the other three environments, with the differences being statistically significant.

Bacteria carrying DRGs are known to exhibit resistance to disinfectants (8). The frequent and high-intensity use of disinfectants in quarantine hotels may selectively promote the survival and proliferation of resistant strains, while more sensitive bacteria are eliminated. This selective pressure facilitates the enrichment of DRGs in the microbial population, contributing to increased disinfectant resistance over time (8). Alternatively, if disinfectants are applied at sublethal concentrations, they may fail to eliminate bacteria effectively, providing further opportunities for microbial adaptation and the propagation of resistance genes (8). The genes *AcrAB*, *OprF*, and *TolC* are closely associated with bacterial resistance mechanisms. Li et al. reported that *AcrAB* and *TolC* together form the AcrAB-TolC efflux pump, which enables bacteria to expel toxic compounds, including disinfectants, from the cell (25). Additionally, Machado et al. demonstrated that *Pseudomonas aeruginosa* adapts to benzalkonium chloride disinfectant through mechanisms involving the *OprF* gene, which encodes an outer membrane protein essential for this resistance (29).

This study similarly found that the highest abundance of ARGs was present in bacteria on the surfaces of elevator buttons in quarantine hotels. The most prevalent ARGs identified in quarantine hotels, infectious disease hospitals, general hospitals, and general hotels were *tetC*, *ermX*, *ermZ*, and *aadA16*, respectively. Disinfectants have been shown to promote the horizontal transfer of ARGs (30).

Residual concentrations of disinfectants can unintentionally select for and enrich an antibiotic resistome through several interrelated mechanisms: (a) Selective pressure at sub-lethal levels, when disinfectant concentrations fall below the minimum bactericidal threshold—either because of dilution over time or uneven application—sensitive cells are killed but more tolerant variants survive. Those surviving variants often carry mutations or mobile elements (e.g., genes encoding efflux pumps) that confer increased tolerance not only to the disinfectant, but also to structurally unrelated antibiotics (cross-resistance) (31); (b) co-selection via mobile genetic elements, many disinfectant-tolerance genes (e.g., qac efflux pumps) reside on the same plasmids or integrons as antibiotic-resistance genes (32); (c) induction of stress responses and enhanced horizontal gene transfer, sub-lethal oxidizing agents (e.g., hypochlorite) can generate reactive oxygen species that trigger the bacterial SOS response. The SOS response upregulates error-prone DNA polymerases (increasing mutation rates) and induces expression of integrase and conjugation machinery, facilitating the acquisition and dissemination of resistance genes (33, 34).

The identified *tetC* from elevator button surfaces in hotel environments aligns with findings from wastewater treatment plant studies, where *tetC* also emerged as a major ARG (35), which encodes resistance to tetracycline antibiotics (36). Zhang et al. found that disinfectant use can affect the abundance of the *tetC* gene, with the *tetC/16S rRNA* ratio increasing in environments with frequent disinfectant exposure (37). Therefore, the elevated abundance of *tetC* on elevator buttons in quarantine hotels is likely related to the high frequency of disinfectant application in these settings.

The abundance of ARGs are influenced primarily by antibiotic usage patterns (35). Both *ermZ* and *ermX* are macrolide resistance genes (38, 39). and their higher prevalence in hospital environments may be attributed to the widespread use of macrolide antibiotics for treating various infections (40). In general hotels, the most prevalent ARG was *aadA16*, which confers resistance to streptomycin and spectinomycin. This gene is commonly found in various pathogens, including *Klebsiella pneumoniae* and *E. coli* (41–43).

Bacteria carrying antibiotic resistance genes (ARGs) can develop resistance to antibiotics (44). In line with the ARG findings of this study, the abundance of antibiotic-resistant bacteria (ARBs) on the surfaces of elevator buttons was highest in quarantine hotels compared to the other three environments. Among the identified pathogens, *Pseudomonas aeruginosa*, *E. coli*, and *Acinetobacter baumannii* showed the highest proportions of antibiotic resistance in quarantine hotels. These three bacteria are clinically significant pathogens known to pose serious risks to human health. Drug-resistant *P. aeruginosa* presents a major challenge in clinical settings, particularly for immunocompromised or chronically ill patients, due to its resistance to multiple antibiotic classes (45). Once considered a relatively low-risk pathogen, *A. baumannii* has attracted increasing attention over the past decade owing to its rapid acquisition and spread of antibiotic resistance (46). In particular, the emergence of carbapenem-resistant *A. baumannii* (CRAB) has had substantial implications for both clinical treatment and public health (47, 48).

In addition to vertical transmission to offspring, bacterial resistance genes can also be transferred between bacteria of the same or different species through horizontal gene transfer (HGT) (48). HGT primarily facilitates the dissemination of resistance genes via mobile genetic elements (MGEs), which are key contributors to the spread of both antibiotic resistance genes (ARGs) and disinfectant resistance genes (DRGs) (49, 50)_._ In this study, bacteria on the surfaces of elevator buttons in quarantine hotels exhibited the highest abundance of MGEs among the four environments examined.

Furthermore, the most frequently detected MGEs across all environments were transposases, followed by the insertion sequence *IS91*. The high frequency of disinfectant use in quarantine hotels may enhance the horizontal transfer of MGEs between bacterial species. Jin et al. reported that chlorine-containing disinfectants can promote plasmid-mediated horizontal gene transfer, enabling intergeneric exchange of ARGs and fostering the emergence of new antibiotic-resistant bacteria (ARBs) (31). Transposases are among the most commonly identified MGEs (51), S Shi et al. also found them to be the most abundant MGE type in pesticide-contaminated wastewater treatment plants (52). *IS91*, often located near virulence-associated genes, plays a significant role in the dissemination and evolution of bacterial pathogenicity and virulence (53).

## 5 Limitations of the study

This study has several limitations that should be acknowledged. First, the sample size and geographic scope were limited, as samples were collected from only ten sites within a single city (Nanjing, China), which may restrict the generalizability of the findings to other regions or environments. Second, the sampling period was relatively short (September to October 2022), preventing assessment of temporal variations or seasonal trends in microbial communities and resistance gene profiles. Third, although metagenomic sequencing enabled detection of antibiotic resistance genes (ARGs), disinfectant resistance genes (DRGs), and mobile genetic elements (MGEs), the study did not validate their functional expression or resistance phenotypes through culture-based assays. Additionally, daily human traffic could influence microbial load. These limitations highlight the need for broader, longitudinal, and functionally integrated studies in future research.

## 6 Conclusions and recommendation

In summary, this study characterized the bacterial community structure on elevator button surfaces across four different environments and identified the presence of potential bacterial pathogens. The frequent disinfection measures implemented during the COVID-19 pandemic may have influenced the prevalence of resistance genes. Our findings showed that the abundance of disinfectant resistance genes (DRGs) and antibiotic resistance genes (ARGs) was significantly higher in quarantine hotels compared to general hospitals, infectious disease hospitals, and general hotels. Additionally, the elevated levels of mobile genetic elements (MGEs) in quarantine hotels suggest a potential mechanism for the spread of DRGs and ARGs in this setting. To maintain microbial ecological balance, limit the transmission of pathogenic bacteria, and protect public health, it is essential to implement rational and targeted disinfection strategies in the post-pandemic era.

## Data Availability

The original contributions presented in the study are included in the article/Supplementary material, further inquiries can be directed to the corresponding authors.

## References

[1] Bchetnia M Girard C Duchaine C Laprise C. The outbreak of the novel severe acute respiratory syndrome coronavirus 2 (Sars-CoV-2): a review of the current global status. J Infect Public Health. (2020) 13:1601–10. doi: 10.1016/j.jiph.2020.07.011, PMID: 32778421 PMC7402212

[2] Akter R Rahman MH Bhattacharya T Kaushik D Mittal V Parashar J. Novel coronavirus pathogen in humans and animals: an overview on its social impact, economic impact, and potential treatments. Environ Sci Pollut Res Int. (2021) 28:68071–89. doi: 10.1007/s11356-021-16809-8, PMID: 34664166 PMC8523003

[3] Gaspari R Spinazzola G Teofili L Avolio AW Fiori B Maresca GM. Protective effect of Sars-CoV-2 preventive measures against Eskape and *Escherichia coli* infections. Eur J Clin Investig. (2021) 51:e13687. doi: 10.1111/eci.13687, PMID: 34599600 PMC8646464

[4] Tregoning JS Flight KE Higham SL Wang Z Pierce BF. Progress of the Covid-19 vaccine effort: viruses, vaccines and variants versus efficacy, effectiveness and escape. Nat Rev Immunol. (2021) 21:626–36. doi: 10.1038/s41577-021-00592-1, PMID: 34373623 PMC8351583

[5] Sadeghi S Kalantari Y Shokri S Fallahpour M Nafissi N Goodarzi A. Immunologic response, efficacy, and safety of vaccines against COVID-19 infection in healthy and immunosuppressed children and adolescents aged 2 – 21 years old: a systematic review and Meta-analysis. J Clin Virol. (2022) 153:105196. doi: 10.1016/j.jcv.2022.105196, PMID: 35716417 PMC9162782

[6] Rai NK Ashok A Akondi BR. Consequences of chemical impact of disinfectants: safe preventive measures against Covid-19. Crit Rev Toxicol. (2020) 50:513–20. doi: 10.1080/10408444.2020.1790499, PMID: 32729370

[7] Mc Carlie S Boucher CE Bragg RR. Corrigendum to "molecular basis of bacterial disinfectant resistance" [drug resist. Updates 48 (2020) 100672]. Drug Resist Updat. (2022) 65:100867. doi: 10.1016/j.drup.2022.100867 36252361

[8] Tong C Hu H Chen G Li Z Li A Zhang J. Disinfectant resistance in bacteria: mechanisms, spread, and resolution strategies. Environ Res. (2021) 195:110897. doi: 10.1016/j.envres.2021.110897, PMID: 33617866

[9] Rutala WA Kanamori H Gergen MF Sickbert-Bennett EE Weber DJ. Susceptibility of Candida auris and *Candida albicans* to 21 germicides used in healthcare facilities. Infect Control Hosp Epidemiol. (2019) 40:380–2. doi: 10.1017/ice.2019.1, PMID: 30767810

[10] Guo J Liao M He B Liu J Hu X Yan D. Impact of the Covid-19 pandemic on household disinfectant consumption behaviors and related environmental concerns: a questionnaire-based survey in China. J Environ Chem Eng. (2021) 9:106168. doi: 10.1016/j.jece.2021.106168, PMID: 34395190 PMC8349428

[11] Wang H Masters S Edwards MA Falkinham JO III Pruden A. Effect of disinfectant, water age, and pipe materials on bacterial and eukaryotic community structure in drinking water biofilm. Environ Sci Technol. (2014) 48:1426–35. doi: 10.1021/es402636u, PMID: 24401122

[12] Lynch JB Hsiao EY. Microbiomes as sources of emergent host phenotypes. Science. (2019) 365:1405–9. doi: 10.1126/science.aay0240, PMID: 31604267

[13] Singh A. Covid-19: disinfectants and sanitisers are changing microbiomes. BMJ. (2020) 370:m2795. doi: 10.1136/bmj.m2795, PMID: 32665219

[14] Khan S Beattie TK Knapp CW. Relationship between antibiotic- and disinfectant-resistance profiles in bacteria harvested from tap water. Chemosphere. (2016) 152:132–41. doi: 10.1016/j.chemosphere.2016.02.086, PMID: 26966812

[15] Usman M Farooq M Hanna K. Environmental side effects of the injudicious use of antimicrobials in the era of Covid-19. Sci Total Environ. (2020) 745:141053. doi: 10.1016/j.scitotenv.2020.141053, PMID: 32702547 PMC7368658

[16] Murray AK. The novel coronavirus Covid-19 outbreak: global implications for antimicrobial resistance. Front Microbiol. (2020) 11:1020. doi: 10.3389/fmicb.2020.01020, PMID: 32574253 PMC7237633

[17] Pan L Wang J Wang X Ji JS Ye D Shen J. Prevention and control of coronavirus disease 2019 (Covid-19) in public places. Environ Pollut. (2022) 292:118273. doi: 10.1016/j.envpol.2021.118273, PMID: 34634404 PMC8498926

[18] Chen B Han J Dai H Jia P. Biocide-tolerance and antibiotic-resistance in community environments and risk of direct transfers to humans: unintended consequences of community-wide surface disinfecting during Covid-19? Environ Pollut. (2021) 283:117074. doi: 10.1016/j.envpol.2021.117074, PMID: 33848900 PMC8019131

[19] Parveen N Chowdhury S Goel S. Environmental impacts of the widespread use of chlorine-based disinfectants during the Covid-19 pandemic. Environ Sci Pollut Res Int. (2022) 29:85742–60. doi: 10.1007/s11356-021-18316-2, PMID: 35091954 PMC8799444

[20] Xie C Zhao H Li K Zhang Z Lu X Peng H. The evidence of indirect transmission of Sars-CoV-2 reported in Guangzhou, China. BMC Public Health. (2020) 20:1202. doi: 10.1186/s12889-020-09296-y, PMID: 32758198 PMC7403788

[21] Zhou J Wang J Zhou Y Liu K Lu Y Zhu L. Microbial community structure and interactions between aspergillus oryzae and bacteria in traditional solid-state fermentation of Jiangqu. Food Microbiol. (2023) 116:104346. doi: 10.1016/j.fm.2023.104346, PMID: 37689429

[22] Wang JL Liu KL Zhao XQ Zhang HQ Li D Li JJ. Balanced fertilization over four decades has sustained soil microbial communities and improved soil fertility and rice productivity in red paddy soil. Sci Total Environ. (2021) 793:148664. doi: 10.1016/j.scitotenv.2021.148664 34328991

[23] Boyce JM. Quaternary ammonium disinfectants and antiseptics: tolerance, resistance and potential impact on antibiotic resistance. Antimicrob Resist Infect Control. (2023) 12:32. doi: 10.1186/s13756-023-01241-z, PMID: 37055844 PMC10099023

[24] Kandel CE Simor AE Redelmeier DA. Elevator buttons as unrecognized sources of bacterial colonization in hospitals. Open Med. (2014) 8:e81–6. 25426176 PMC4242253

[25] Li H Li X Chen T Yang Z Shi D Yin J. Antidepressant exposure as a source of disinfectant resistance in waterborne bacteria. J Hazard Mater. (2023) 452:131371. doi: 10.1016/j.jhazmat.2023.131371, PMID: 37030229

[26] Pereira Da Fonseca TA Pessôa R Felix AC Sanabani SS. Diversity of bacterial communities on four frequently used surfaces in a large Brazilian teaching hospital. Int J Environ Res Public Health. (2016) 13:152. doi: 10.3390/ijerph13020152, PMID: 26805866 PMC4772172

[27] Adams RI Bhangar S Pasut W Arens EA Taylor JW Lindow SE. Chamber bioaerosol study: outdoor air and human occupants as sources of indoor airborne microbes. PLoS One. (2015) 10:e0128022. doi: 10.1371/journal.pone.0128022, PMID: 26024222 PMC4449033

[28] Guo YR Cao QD Hong ZS Tan YY Chen SD Jin HJ. The origin, transmission and clinical therapies on coronavirus disease 2019 (Covid-19) outbreak - an update on the status. Mil Med Res. (2020) 7:11. doi: 10.1186/s40779-020-00240-0, PMID: 32169119 PMC7068984

[29] Machado I Coquet L Jouenne T Pereira MO. Proteomic approach to *Pseudomonas aeruginosa* adaptive resistance to benzalkonium chloride. J Proteome. (2013) 89:273–9. doi: 10.1016/j.jprot.2013.04.030, PMID: 23651563

[30] Maillard JY Pascoe M. Disinfectants and antiseptics: mechanisms of action and resistance. Nat Rev Microbiol. (2024) 22:4–17. doi: 10.1038/s41579-023-00958-3, PMID: 37648789

[31] Jin M Liu L Wang DN Yang D Liu WL Yin J. Chlorine disinfection promotes the exchange of antibiotic resistance genes across bacterial genera by natural transformation. ISME J. (2020) 14:1847–56. doi: 10.1038/s41396-020-0656-9, PMID: 32327733 PMC7305130

[32] Murray LM Hayes A Snape J Kasprzyk-Hordern B Gaze WH Murray AK. Co-selection for antibiotic resistance by environmental contaminants. NPJ Antimicrob Resist. (2024) 2:9. doi: 10.1038/s44259-024-00026-7, PMID: 39843965 PMC11721650

[33] Zhang S Wang Y Lu J Yu Z Song H Bond PL. Chlorine disinfection facilitates natural transformation through Ros-mediated oxidative stress. ISME J. (2021) 15:2969–85. doi: 10.1038/s41396-021-00980-4 33941886 PMC8091644

[34] Liu S-S Qu HM Yang D Hu H Liu WL Qiu ZG. Chlorine disinfection increases both intracellular and extracellular antibiotic resistance genes in a full-scale wastewater treatment plant. Water Res. (2018) 136:131–6. doi: 10.1016/j.watres.2018.02.036, PMID: 29501757

[35] Wang Y Han Y Li L Liu J Yan X. Distribution, sources, and potential risks of antibiotic resistance genes in wastewater treatment plant: a review. Environ Pollut. (2022) 310:119870. doi: 10.1016/j.envpol.2022.119870 35921944

[36] Kadlec K Schwarz S. Antimicrobial resistance in *Bordetella bronchiseptica*. Microbiol Spectr. (2018) 6:10-1128. doi: 10.1128/microbiolspec.ARBA-0024-2017, PMID: 30027886 PMC11633599

[37] Zhang Y Li A Dai T Li F Xie H Chen L. Cell-free Dna: a neglected source for antibiotic resistance genes spreading from Wwtps. Environ Sci Technol. (2018) 52:248–57. doi: 10.1021/acs.est.7b04283, PMID: 29182858

[38] Hächler H Berger-Bächi B Kayser FH. Genetic characterization of a *Clostridium difficile* erythromycin-clindamycin resistance determinant that is transferable to *Staphylococcus aureus*. Antimicrob Agents Chemother. (1987) 31:1039–45. doi: 10.1128/AAC.31.7.1039, PMID: 2821888 PMC174868

[39] El Damaty HM El-Demerdash AS Abd El-Aziz NK Yousef SG Hefny AA Abo Remela EM. Molecular characterization and antimicrobial susceptibilities of *Corynebacterium pseudotuberculosis* isolated from Caseous lymphadenitis of smallholder sheep and goats. Animals (Basel). (2023) 13:2337. doi: 10.3390/ani13142337, PMID: 37508114 PMC10376069

[40] Nater A Von Garnier C Mouraux S. Macrolides antibiotics and chronic pulmonary disease. Rev Med Suisse. (2022) 18:2206–12. doi: 10.53738/REVMED.2022.18.805.2206, PMID: 36416507

[41] Sun J Zhou M Wu Q Ni Y. Characterization of two novel gene cassettes, dfrA27 and aadA16, in a non-O1, non-O139 *Vibrio cholerae* isolate from China. Clin Microbiol Infect. (2010) 16:1125–9. doi: 10.1111/j.1469-0691.2009.03060.x, PMID: 19906273

[42] Huang L Fu L Hu X Liang X Gong G Xie C. Co-occurrence of Klebsiella variicola and *Klebsiella pneumoniae* both carrying Bla (Kpc) from a respiratory intensive care unit patient. Infect Drug Resist. (2021) 14:4503–10. doi: 10.2147/IDR.S330977, PMID: 34744441 PMC8565889

[43] Stoesser N Batty EM Eyre DW Morgan M Wyllie DH del Ojo Elias C. Predicting antimicrobial susceptibilities for *Escherichia coli* and *Klebsiella pneumoniae* isolates using whole genomic sequence data. J Antimicrob Chemother. (2013) 68:2234–44. doi: 10.1093/jac/dkt180, PMID: 23722448 PMC3772739

[44] Jian Z Zeng L Xu T Sun S Yan S Yang L. Antibiotic resistance genes in bacteria: occurrence, spread, and control. J Basic Microbiol. (2021) 61:1049–70. doi: 10.1002/jobm.202100201 34651331

[45] Diggle SP Whiteley M. Microbe profile: *Pseudomonas aeruginosa*: opportunistic pathogen and lab rat. Microbiology (Reading). (2020) 166:30–3. doi: 10.1099/mic.0.000860, PMID: 31597590 PMC7273324

[46] Chakravarty B. Genetic mechanisms of antibiotic resistance and virulence in *Acinetobacter baumannii*: background, challenges and future prospects. Mol Biol Rep. (2020) 47:4037–46. doi: 10.1007/s11033-020-05389-4, PMID: 32303957

[47] Paitan Y. Current trends in antimicrobial resistance of *Escherichia coli*. Curr Top Microbiol Immunol. (2018) 416:181–211. doi: 10.1007/82_2018_110, PMID: 30088148

[48] Yadav S Kapley A. Exploration of activated sludge resistome using metagenomics. Sci Total Environ. (2019) 692:1155–64. doi: 10.1016/j.scitotenv.2019.07.267, PMID: 31539947

[49] Wiedenbeck J Cohan FM. Origins of bacterial diversity through horizontal genetic transfer and adaptation to new ecological niches. FEMS Microbiol Rev. (2011) 35:957–76. doi: 10.1111/j.1574-6976.2011.00292.x, PMID: 21711367

[50] Partridge SR Kwong SM Firth N Jensen SO. Mobile genetic elements associated with antimicrobial resistance. Clin Microbiol Rev. (2018) 31:e00088-17. doi: 10.1128/CMR.00088-17, PMID: 30068738 PMC6148190

[51] Jiao X Guo W Li X Yao F Zeng M Yuan Y. New insight into the microbiome, resistome, and mobilome on the dental waste water in the context of heavy metal environment. Front Microbiol. (2023) 14:1106157. doi: 10.3389/fmicb.2023.1106157, PMID: 37152760 PMC10157219

[52] Shi L Zhang J Lu T Zhang K. Metagenomics revealed the mobility and hosts of antibiotic resistance genes in typical pesticide wastewater treatment plants. Sci Total Environ. (2022) 817:153033. doi: 10.1016/j.scitotenv.2022.153033, PMID: 35026253

[53] Garcillán-Barcia MP De La Cruz F. Distribution of Is91 family insertion sequences in bacterial genomes: evolutionary implications. FEMS Microbiol Ecol. (2002) 42:303–13. doi: 10.1111/j.1574-6941.2002.tb01020.x, PMID: 19709290

