# Supplementary material for: Microbial community structure and resistome dynamics on elevator buttons in response to surface disinfection practices
Source: Front Public Health. 2025 May 30;13:1593114. doi: 10.3389/fpubh.2025.1593114 (PMC12162647; doi:10.3389/fpubh.2025.1593114)
Supplement: Supplementary file 1 [file Data_Sheet_1.docx]

Supplementary Materials

Microbial community structure and resistome dynamics on elevator buttons in response to surface disinfection practices

Shanshan Ye^1,2†^, Shifu Peng^1†^, Xiaolei Wang^1,3^, Jingjing Fan^1,3^, Chenxue Zhu^2^, Liye Huang^4^, Ying Huang^5^, Keping Cheng^6^, Tingting Ni^3^, Yuqing Qian^1^, Xiaosong Wu^1,2,3,7*^, Yan Xu^1,2,3,7*^

^1^ Jiangsu Provincial Center for Disease Control and Prevention, Nanjing, Jiangsu, China.

^2^ School of Public Health, Nanjing Medical University, Nanjing, Jiangsu, China.

^3^ National Health Committee Key Laboratory of Enteric Pathogenic Microbiology, Nanjing, Jiangsu, China.

^4^ Xuzhou Center for Disease Control and Prevention, Xuzhou, Jiangsu, China.

^5^ Infection Control Department, The Second Hospital of Nanjing, Affiliated to Nanjing University of Chinese Medicine, Nanjing, Jiangsu, China.

^6^ Zhongda Hospital Affiliated to Southeast University, Nanjing, Jiangsu, China.

^7^ Jiangsu Provincial Medical Key Laboratory of Pathogenic Microbiology in Emerging Major Infectious Diseases, Nanjing, Jiangsu, China.

^†^ These authors have contributed equally to this work.

^⁎^ Correspondence to [cdcxy@vip.sina.com](mailto:cdcxy@vip.sina.com) (Y. Xu) and sxwbox@163.com (X. Wu)


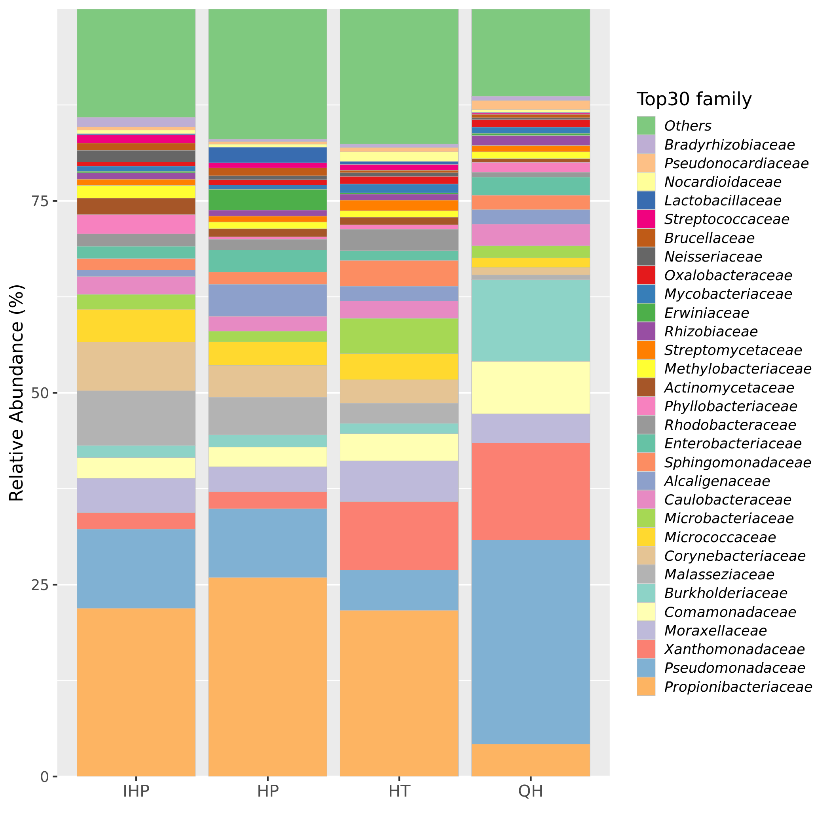


**FIGURE S1.** Distribution and absolute abundance of the top 30 bacterial families in four different environments. IHP, HP, HT, and QH represent infectious disease hospital, general hospitals, general hotels, and quarantine hotels, respectively.


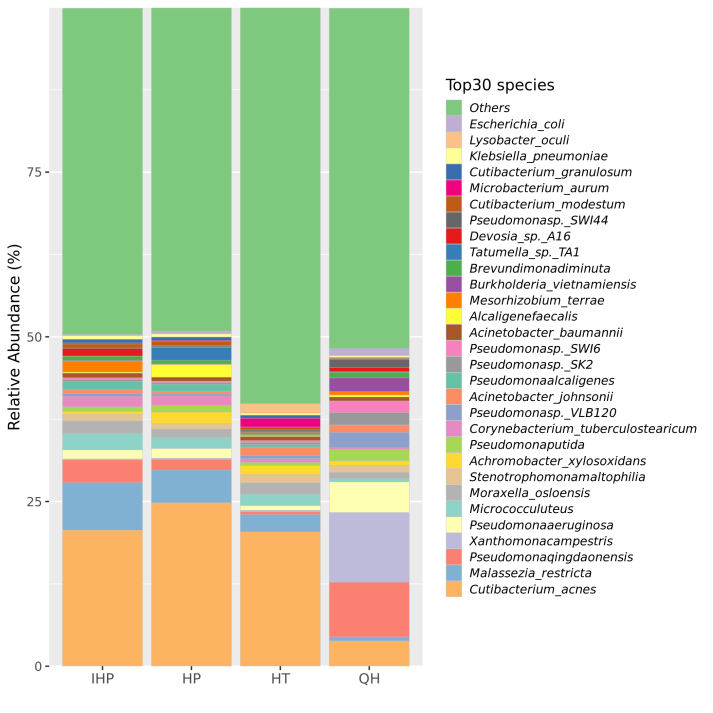


**FIGURE S2.** Distribution and absolute abundance of the top 30 bacterial species in four different environments. IHP, HP, HT, and QH represent infectious disease hospital, general hospitals, general hotels, and quarantine hotels, respectively.


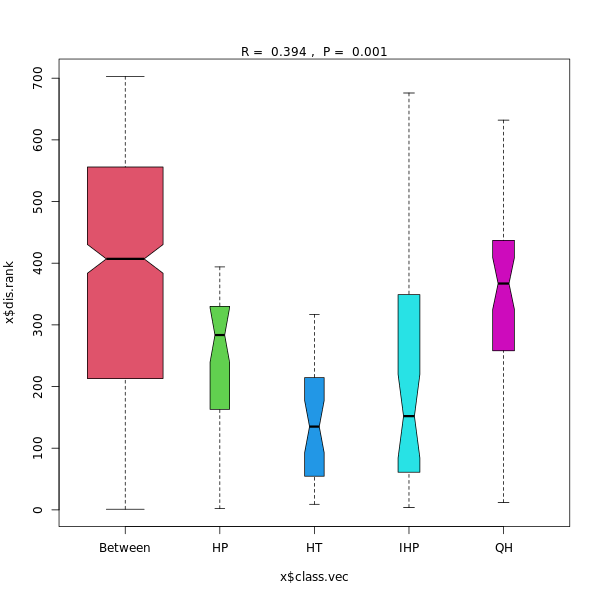


**FIGURE S3.** Similarity analysis for Bray–Curtis heterogeneity test (ANOSIM). IHP, HP, HT, and QH represent infectious disease hospital, general hospitals, general hotels, and quarantine hotels, respectively.


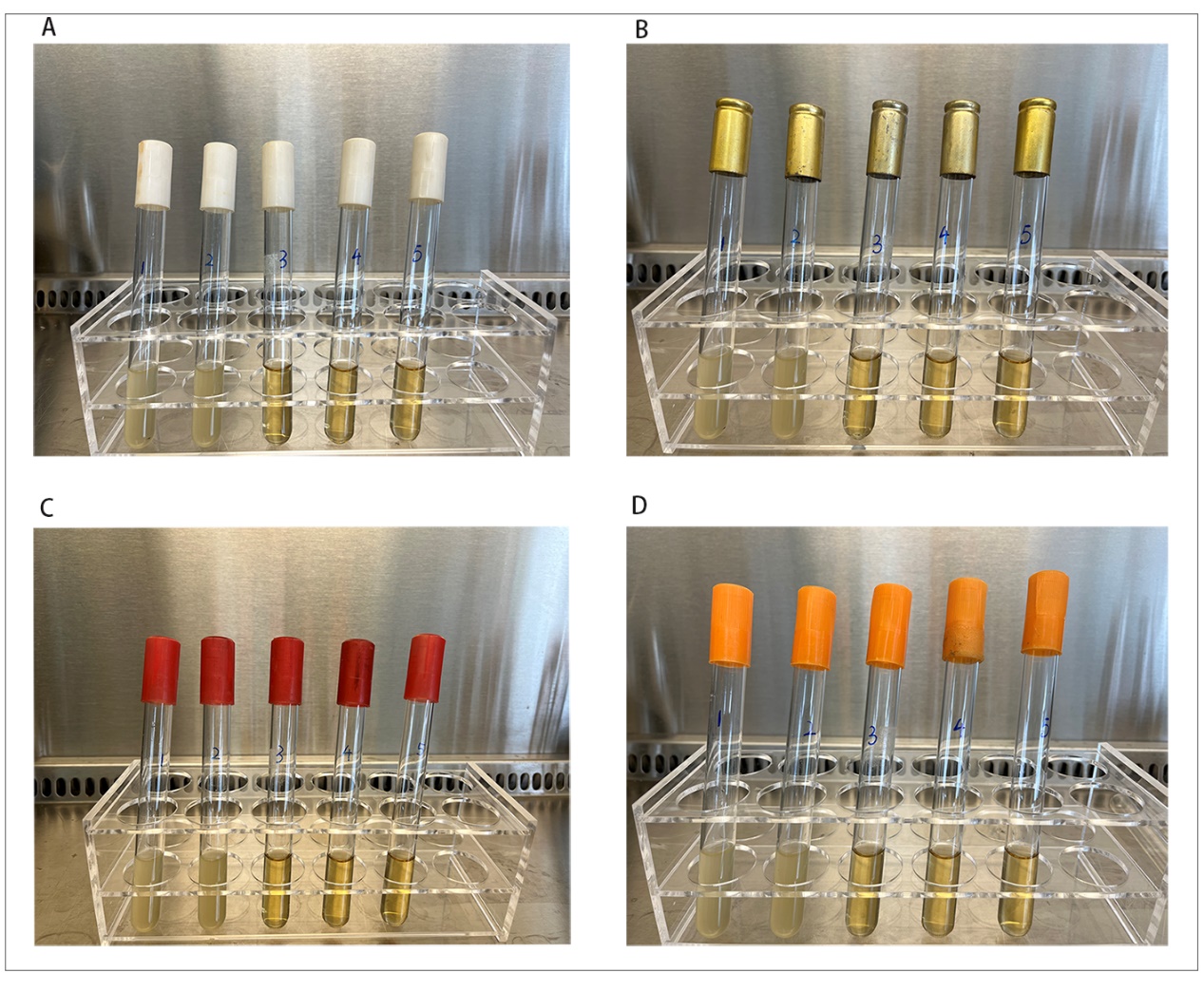


**FIGURE S4.** The MIC experiment results of four strains of *S. aureus*. The MIC values of all four strains of Staphylococcus aureus were 200 mg/L. The concentration of chlorine-containing disinfectant in five test tubes (from left to right) was 100 mg/L,150 mg/L, 200 mg/L, 250 mg/L, and 300 mg/L. Turbidity indicates bacterial growth, while clear indicates no bacterial growth. (A), (B), (C), and (D) represents the MIC results of *S. aureus* isolated from quarantine hotels, general hotels, general hospitals, and an infectious disease hospital. Turbidity was observed in the first tube and the second tube.


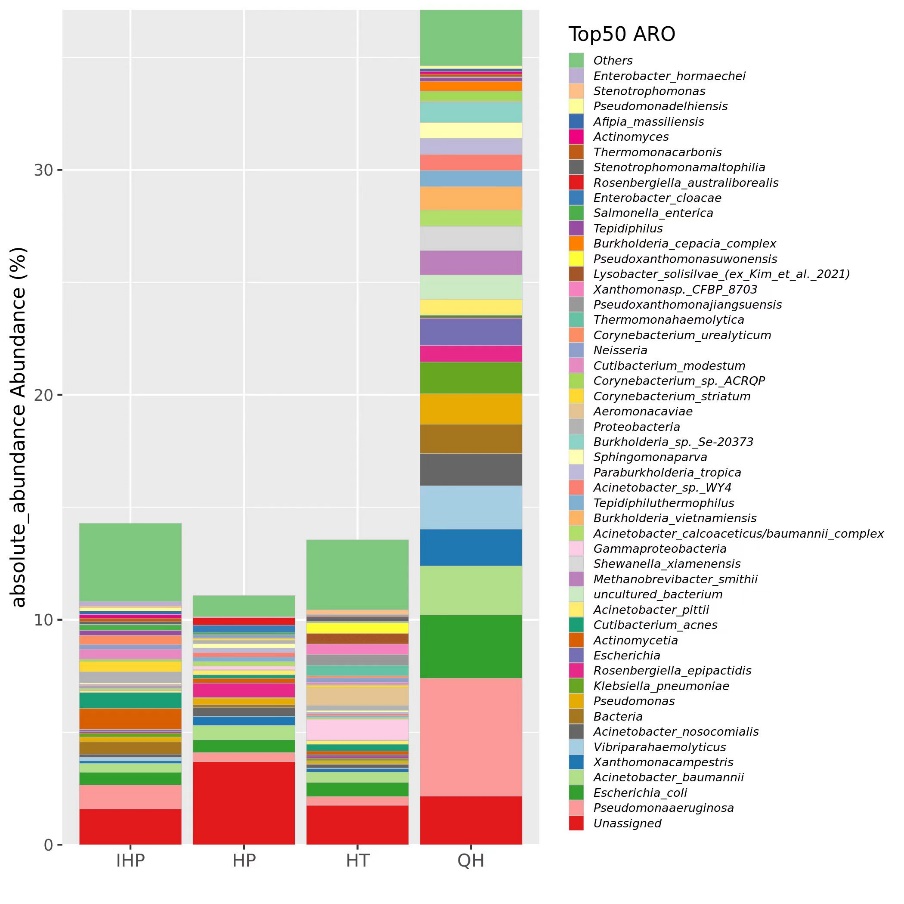

**FIGURE S5.** Species and absolute abundance of the top 50 resistant bacteria in four different environments. IHP, HP, HT, and QH represent infectious disease hospital, general hospitals, general hotels, and quarantine hotels, respectively.


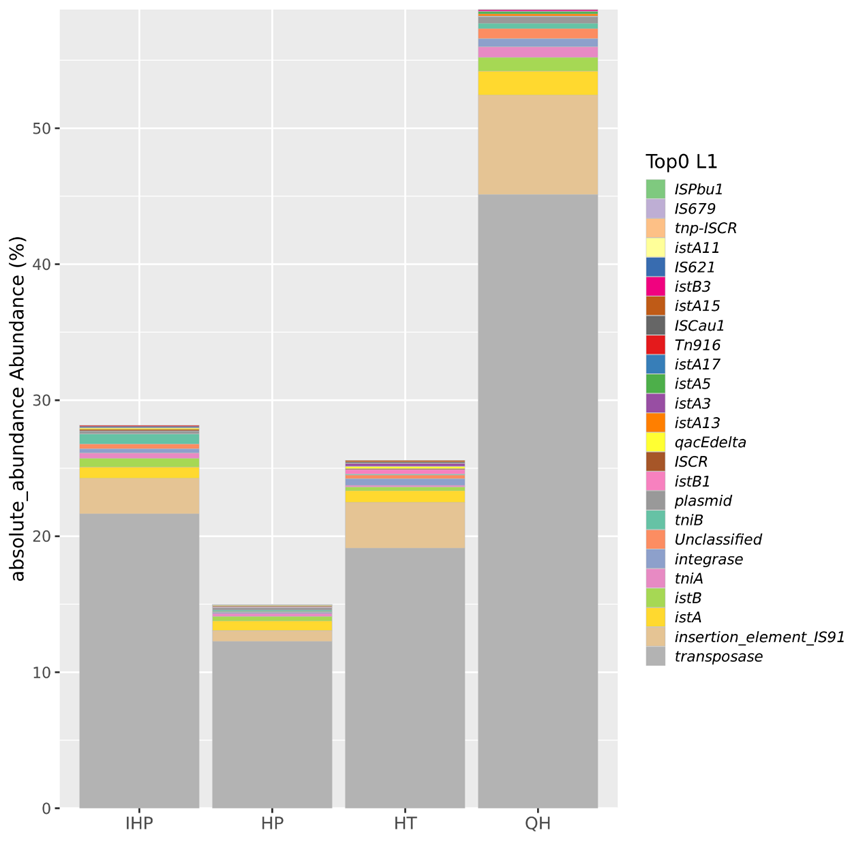


**Fig. S6.** The species and absolute abundance of mobile genetic elements in four environments. IHP, HP, HT, and QH represent infectious disease hospital, general hospitals, general hotels, and quarantine hotels, respectively.
